# The productivity gains associated with a junk food tax and their impact on cost-effectiveness

**DOI:** 10.1371/journal.pone.0220209

**Published:** 2019-07-22

**Authors:** Hannah E. Carter, Deborah J. Schofield, Rupendra Shrestha, Lennert Veerman

**Affiliations:** 1 Australian Centre for Health Services Innovation, Institute of Health and Biomedical Innovation, School of Public Health and Social Work, Faculty of Health, Queensland University of Technology, Brisbane, Queensland, Australia; 2 Centre for Economic Impacts of Genomic Medicine, Macquarie University, Sydney, New South Wales, Australia; 3 School of Medicine, Griffith University, Gold Coast, Queensland, Australia; National Institute of Health and Nutrition, National Institutes of Biomedical Innovation, Health and Nutrition, JAPAN

## Abstract

**Objective:**

To estimate the productivity impacts of a policy intervention on the prevention of premature mortality due to obesity.

**Methods:**

A simulation model of the Australian population over the period from 2003 to 2030 was developed to estimate productivity gains associated with premature deaths averted due to an obesity prevention intervention that applied a 10% tax on unhealthy foods. Outcome measures were the total working years gained, and the present value of lifetime income (PVLI) gained. Impacts were modelled over the period from 2003 to 2030. Costs are reported in 2018 Australian dollars and a 3% discount rate was applied to all future benefits.

**Results:**

Premature deaths averted due to a junk food tax accounted for over 8,000 additional working years and a $307 million increase in PVLI. Deaths averted in men between the ages of 40 to 59, and deaths averted from ischaemic heart disease, were responsible for the largest gains.

**Conclusions:**

The productivity gains associated with a junk food tax are substantial, accounting for almost twice the value of the estimated savings to the health care system. The results we have presented provide evidence that the adoption of a societal perspective, when compared to a health sector perspective, provides a more comprehensive estimate of the cost-effectiveness of a junk food tax.

## Introduction

Overweight and obesity are well established risk factors for a number of chronic diseases including cardiovascular diseases, cancers and diabetes [1, 2]. The rise in sedentary lifestyles and the increased consumption of energy dense foods has seen worldwide obesity rates more than double since 1980, with the prevalence of chronic disease increasing globally across every region [3, 4]. As a result, overweight and obesity are increasingly recognised as being among the most important public health issues in the world today [5].

The most recent Australian Health Survey highlights that 28% of adults are now obese, with 63% classified as overweight or obese [6]. Projections suggest that by 2025, the prevalence of overweight and obesity will increase to over 70%, with approximately one third of the adult Australian population classified as obese [7]. Reflecting a similar circumstance globally, WHO member states have introduced a voluntary target to halt the rise in obesity by 2020 [8].

In addition to its significant health burden, obesity is also responsible for a substantial economic burden. The cost of illness framework provides an approach for estimating the economic burden of disease that incorporates both direct health care resource use, as well as the indirect productivity impacts of illness and death [9]. Such studies are well represented in the medical literature around overweight and obesity, and it has been reported that the direct health care costs associated with these risk factors are significant, accounting for between 2–12% of total health care budgets in developed economies [10–15]. Where these studies have considered the productivity related costs of overweight and obesity, these costs have consistently been found to outweigh the direct health care costs [16–18].

Despite these findings, studies evaluating the cost-effectiveness of public health interventions typically take a health care perspective, with estimates of potential cost savings limited to those associated with health care resource use. The cost-effectiveness outcomes reported in these studies may underestimate the total benefits to society. It follows that decisions regarding the allocation of society’s scare resources towards improving health are often made without full information on the potential economic returns of these investments.

The aim of this study was to estimate the productivity impacts of a tax that would raise prices on unhealthy foods by 10% in an Australian setting. The selected unhealthy food categories included biscuits, cakes, pastries, pies, snack foods, confectionary and soft drinks. We refer to this intervention throughout as the ‘junk food tax’. Our secondary aim was to determine how the inclusion of productivity impacts of a junk food tax influenced the overall cost-effectiveness outcome. We applied microsimulation techniques to project the lifetime working years and income that would accrue for individuals whose premature deaths could be successfully averted under the intervention. Outcomes were modelled to the year 2030 and are presented across a number of age, sex and disease categories.

## Methods

**Fig 1:**
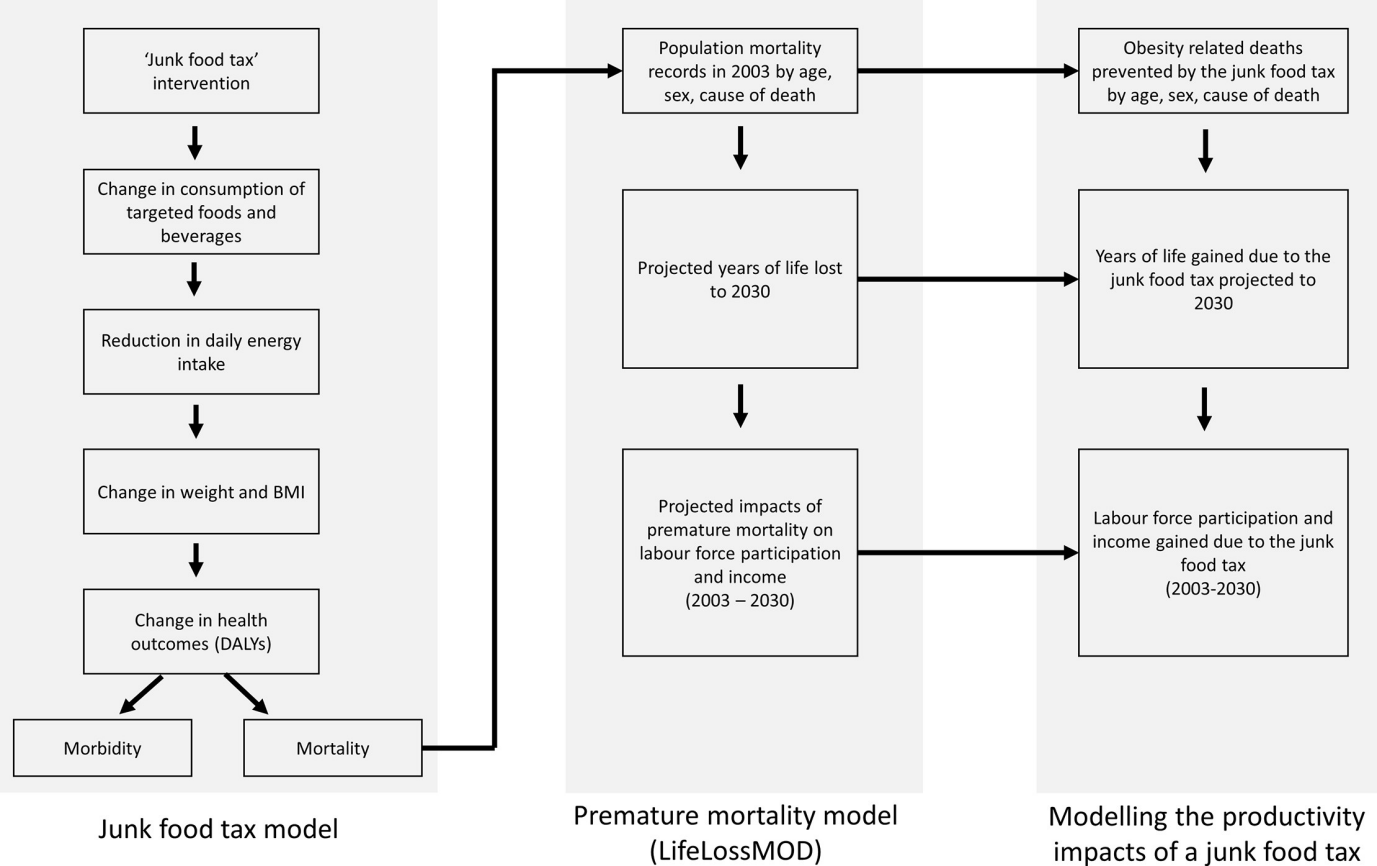
Logic pathways for modelling the effect of a junk food tax on economic productivity due to premature deaths averted.

This study combines data from two previously published models in order to estimate the productivity gains associated with a junk food tax [19, 20]. Fig 1 outlines the logic pathways that guide each model and their intersections with one another. The analysis is based on an Australian adult population, aged 20 and above. Premature mortality was defined here as deaths occurring before the age of 80 years, which is close to the Australian life expectancy [21, 22].

An estimate of the number of Disability Adjusted Life Years (DALYs) averted due to the junk food tax was estimated by Sacks et al (2011) [19, 23]. The authors developed a Markov model to project the impact of changes in BMI on a number of obesity-related diseases, including: stroke, ischaemic heart disease, hypertensive heart disease, diabetes mellitus, osteoarthritis, post-menopausal breast cancer, colon cancer, endometrial cancer and kidney cancer. The model compared two populations in separate life tables: a baseline population that was exposed to existing levels of morbidity and mortality, and an intervention population where, owing to lower average body weight, the risk of disease was reduced. Baseline consumption of unhealthy foods was based on the latest available food consumption data for the Australian adult population, from the 1995 National Nutrition Survey [24]. Changes in junk food consumption following the imposition of the junk food tax were estimated at an individual level. These changes in energy consumption were then extrapolated to project changes in mean body weight and BMI at the population level, using equations derived by Swinburn et al [25]. For the purpose of this study, we extracted estimates of the change in the number of deaths across combinations of age, sex and cause of death from the original model.

Estimates of changes in mortality due to the junk food tax were then combined with data from LifeLossMOD, a separate model developed to estimate the productivity costs associated with all-cause premature mortality [20]. LifeLossMOD was developed using individual level mortality data from the 2003 Australian Burden of Disease and Injury study [26]. The model then applied microsimulation methods to assign a counterfactual life trajectory to each individual that died in 2003. These trajectories were assumed to reflect a likely (albeit hypothetical) scenario that would occur if an individual’s premature death in 2003 had been prevented. Individuals within the model were then tracked to the year 2030, with the model annually updating data on hours worked and income earned. The model accounted for individual variability in age, sex and socio-economic status at the time of death in estimating the counterfactual labour force participation rates, earnings, retirement ages and age at death.

A random process with replacement was used to link each death averted due to the junk food tax with an individual of the same age category, sex and cause of death in LifeLossMOD. To align with the LifeLossMOD estimates, it was assumed that the intervention commenced in the year 2003 and was sustained until the year 2030. Premature deaths could be averted at any point up to 25 years after the beginning of the intervention. A new dataset of premature deaths averted was then created, with each death averted assigned the life trajectory of an individual of the same age, sex and socioeconomic status in LifeLossMOD. Associated information on the annual number of hours worked and income earned until the year 2030 was accrued on an annual basis, with a discount rate of 3% applied to all future impacts.

It was then possible to derive estimates of the additional working years gained due to the intervention by summing the hours worked in each year following a death averted. The resulting figure was then divided by the number of hours in a standard working year (1,976 hours) to produce an estimate of the ‘full-time equivalent’ working years gained for each individual over the period 2003–2030.

To estimate the value of the productivity gains associated with the intervention, we adopted the human capital approach [27]. This method involves valuing the productivity impact of mortality as the present value of the lifetime stream of all future income that would have been earned in the event that a premature death was avoided. While other valuation methods exist, the human capital approach is the most common approach for valuing productivity losses in the cost of illness literature [28–30]. For the purpose of this study, the productivity gains associated with the intervention were therefore estimated by calculating the present value of lifetime income (PVLI) earned by each individual whose death was averted. Income was assumed to come from salary-related earnings, as well as business profits and other investments. However, transfer payments were excluded from this analysis to avoid a ‘double-counting’ of savings at the societal level. Incomes were assumed to grow at a rate of 1% per annum above inflation reflecting long term Australian wage growth trends [31]. All PVLI figures are reported in 2018 Australian dollars.

The process described above was bootstrapped 100 times to incorporate the combined uncertainty across both LifeLossMOD and the junk food tax model. The results presented here describe the mean of the 100 bootstraps, with confidence intervals calculated using the percentile method.

The estimated PVLI savings associated with a junk food tax were combined with data from a previously published cost effectiveness analysis of this intervention [19], conducted from the perspective of the health care system. The impact of productivity costs on the final cost effectiveness outcome was assessed by comparing the results of 100 Monte Carlo simulations of the incremental cost-effectiveness ratio (ICER) under each scenario.

## Results

There were 2,053 premature deaths averted over the first 25 years following the introduction of the junk food tax, of which 71% were among males (Table 1). This translated to 17,403 years of life gained over the period 2003 to 2030. Deaths averted due to ischaemic heart disease accounted for the greatest number of years of life gained (41%), followed by stroke, and diabetes mellitus (18% each).

**Table 1:** Premature deaths averted and associated years of life gained due to the imposition of a junk food tax, modelled from 2003 to 2030.

	Male		Female		Total	
	Premature deaths averted	Years of Life Gained	Premature deaths averted	Years of Life Gained	Premature deaths averted	Years of Life Gained
Ischaemic Heart Disease	691	6,024	150	1,191	841	7,215
*95% CI*	*608–763*	*5*,*279–6*,*632*	*119–181*	*938–1*,*466*	*758–918*	*6*,*455–7*,*815*
Stroke	251	2,046	139	1,124	390	3,170
*95% CI*	*202–299*	*1*,*626–2*,*383*	*106–172*	*838–1*,*494*	*323–455*	*2*,*545–3*,*671*
Diabetes Mellitus	286	2,286	104	821	390	3,107
*95% CI*	*230–339*	*1*,*730–2*,*917*	*86–127*	*566–1*,*101*	*323–445*	*2*,*473–3*,*823*
Colorectal Cancer	113	1,072	47	441	160	1,513
*95% CI*	*91–133*	*857–1*,*335*	*37–57*	*300–561*	*137–180*	*1*,*250–1*,*808*
Hypertensive Heart Disease	71	611	46	405	118	1,016
*95% CI*	*59–87*	*436–795*	*35–56*	*248–559*	*99–137*	*744–1*,*267*
Breast Cancer	-	-	69	649	69	649
*95% CI*	*-*	*-*	*60–76*	*547–747*	*60–76*	*547–747*
Kidney Cancer	52	459	15	122	66	581
*95% CI*	*46–58*	*329–569*	*43040*	*63–182*	*60–74*	*484–682*
Endometrial Cancer	-	-	18	152	18	152
*95% CI*	*-*	*-*	*15–21*	*72–215*	*15–21*	*72–215*
Total	1,464	12,499	588	4,904	2,053	17,403
*95% CI*	*1*,*355–1*,*580*	*11*,*514–13*,*340*	*545–642*	*4*,*396–5*,*520*	*1*,*941–2*,*197*	*16*,*456–18*,*415*

CI = confidence interval

The cumulative working years and PVLI gained between 2003 and 2030 were projected for each death averted (Table 2). A total of 8,656 full time equivalent working years were gained under the modelled scenario which was estimated to provide an addition $307 million in PVLI. Over half of this productivity gain was attributable to male deaths averted between the ages of 40 to 59, consistent with the proportion of years of life gained in this cohort as well as the higher relative incomes among men of this age [31]. Working years gained in those aged 65 and above were possible due to data based projections around the likelihood of individuals working beyond the traditional retirement age.

**Table 2:** Cumulative working years and PVLI gained due to the imposition of a junk food tax: 2003–2030.

Cohort	Premature deaths averted	Working years gained	PVLI gained ($000’s)	95% CI around PVLI gained ($000’s)	PVLI as a proportion of cohort	PVLI gained per death averted ($000’s)
**Men**						
20–29	46	147	7,878	4,874–10,833	3%	171
30–39	173	799	34,298	26,305–43,758	11%	198
40–49	377	2,036	73,403	61,253–84,340	24%	195
50–59	482	2,714	97,510	82,387–117,727	32%	202
60–69	307	889	33,535	25,838–41,420	11%	109
70–79	81	91	1546	343–3,201	1%	19
Total	1,464	6,675	248,170	220,670–272,363	81%	170
**Women**						
20–29	13	43	1,422	556–2,607	0.5%	109
30–39	42	184	5,845	3,800–8,108	2%	139
40–49	102	420	12,612	8,613–16,428	4%	124
50–59	176	775	23,074	18,172–29,036	8%	131
60–69	177	481	14,353	9,390–19,911	5%	81
70–79	78	78	1,117	43–2,404	0.4%	14
Total	588	1,980	58,424	49,275–73,392	19%	99
**Total**	2,053	8,656	306,593	281,724–338,322	100%	149

PVLI = Present value of lifetime income; CI = confidence interval

**Fig 2:**
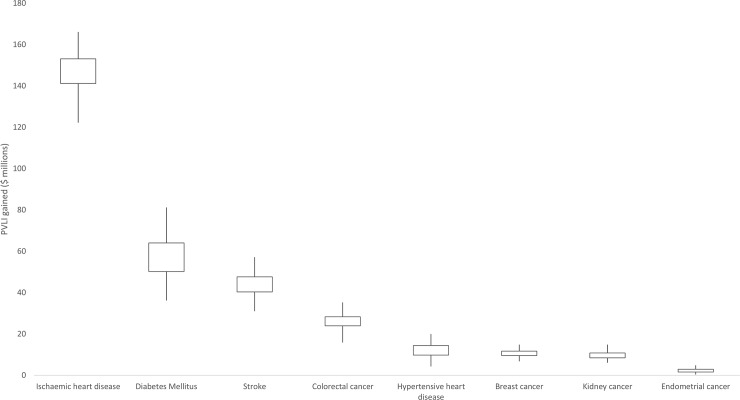
Cumulative PVLI gained from the junk food tax across major cause of death categories (2003 to 2030). Boxes represent the interval between the 25th and 75th percentiles, vertical lines represent the interval between the minimum and maximum observations.

When examining the productivity impacts across disease types, the PVLI gained was broadly consistent with the number of premature deaths averted (Fig 2). Deaths averted from ischaemic heart disease had the largest impact, accounting for $148 million and close to half of the total PVLI gained. Deaths averted from diabetes ($57 million), stroke ($43 million) and colorectal cancer ($26 million) also produced relative large productivity gains.

**Fig 3:**
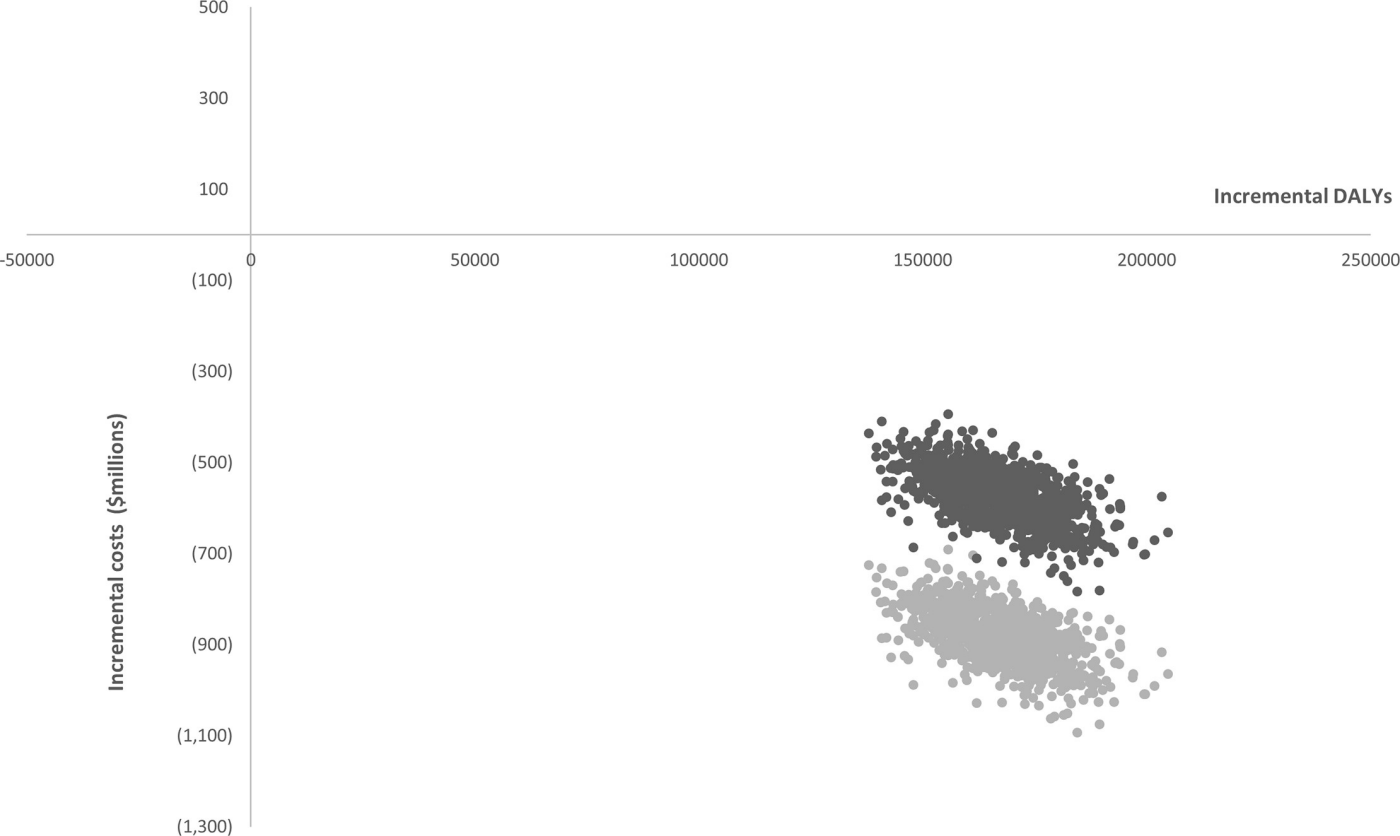
Bootstrapped incremental cost-effectiveness ratio plots for the junk food tax relative to status quo. Dark points represent pairs of incremental costs saved and DALYs averted due the junk food tax based on a health system perspective. Light points represent these points after production gains have been included.

The cost effectiveness of the junk food tax, relative to the status quo, was considered both with and without the inclusion of productivity costs. The estimated savings due to increased productivity ($307 million) amounted to approximately 50% of the savings estimated to fall within the health care system alone ($604 million). As demonstrated by the downwards shift in the ICER estimates in Fig 3 there was a significant improvement in the cost effectiveness outcome when productivity costs were included, with greater uncertainty around cost estimates.

The full datasets used in the analysis have been aggregated by age, sex and cause of death and are included as Supporting Information (S1–S3 Tables).

## Discussion

This paper presents the results of a counterfactual analysis where a junk food tax reduces premature deaths and increases labour force participation and incomes in Australia. Results were modelled to the year 2030. We found the intervention to be associated with a cumulative productivity gain of over 8,000 working years and $307 million in PVLI. The proportion of productivity gain across age, sex and disease categories were broadly consistent with the numbers of deaths averted in each category.

We also demonstrated the impact that the perspective taken can have on the cost effectiveness result. When a societal perspective was taken, with the inclusion of the productivity gains expected to accrue due to improved health from the intervention, the cost effectiveness result was significantly improved. Given the junk food tax was found to be cost-saving even when adopting the health system perspective, the inclusion of productivity costs did not change the overall ‘dominant’ outcome in this case (that is, the intervention was found to be both more effective and less expensive than current practice under both health care system and societal perspectives). However, the magnitude of the downward shift seen on the cost-effectiveness plane clearly demonstrates the potential for the inclusion of productivity costs to influence decisions where incremental cost effectiveness ratios (ICERs) produced under a health system perspective are above the cost effectiveness acceptability threshold.

Taxes on unhealthy foods and drinks have been implemented over the past decade in countries including Denmark, Hungary, the United Kingdom, the United States and Mexico, with promising early results [32–34]. While the longer term population impacts of these policies are yet to be observed, researchers have developed models to project both health and economic outcomes [35–37]. Our results support a growing body of evidence from modelled analyses based on similar underlying methods around the cost-saving nature of taxes on unhealthy foods and drinks [38–40]. While previous studies have focussed on savings to the health system, there has been relatively little published evidence on the productivity impacts. Nomaguchi et al (2017) estimated the productivity impacts of a 20% tax on sugar sweetened beverages and reported an AU $751 million potential gain within the paid employment sector [41]. The larger nature of this estimate relative to our finding may be explained by the inclusion of reductions in both morbidity and mortality in the productivity calculation. The scale of these potential savings suggests that the inclusion of productivity costs in future cost effectiveness analyses of obesity prevention interventions should be considered, particularly when the decision maker values this information.

As obesity rates increase, and more evidence comes to light regarding the impacts of obesity on the prevalence of chronic disease, estimates of the economic burden of obesity can also be expected to increase. While the direct health care costs of obesity are significant and well-recognised, there is an increasing recognition of the indirect costs, in particular those relating to lost economic productivity. A 2014 systematic review found that in studies estimating both the direct and indirect costs of obesity, the productivity related costs consistently outweighed the direct health care costs (accounting for between 51% -59% of the total costs) [16].

The analysis we have presented provides rigorous, population-based estimates of the likely gains in productivity that would accrue with a junk food tax. A key strength of our approach is the projection of long term counterfactual outcomes based on individual characteristics at the time of death. In addition, our estimates allow for projected trends in labour force participation, income levels and retirement ages.

There are some limitations to note. Firstly, this study does not account for the productivity impacts associated with reduced obesity related morbidity. These impacts were excluded due to the lack of available data, with LifeLossMOD developed to estimate to productivity costs of mortality alone. Previous studies have suggested that mortality costs represent approximately two thirds of the total productivity related costs of obesity [42, 43]. We have also excluded productivity gains associated with unpaid labour from this analysis. This is a common practice in the estimation of productivity costs of illness and reflects the definition of productivity as applied in the calculation of national Gross Domestic Product.

Health care budgets across developed countries are increasingly constrained due to the effects of population ageing and ever-advancing medical technology. There is, therefore, an imperative for governments to make decisions about the allocation of society’s scarce resources with a knowledge of the full extent of the returns on investment that can be expected. In this context, the productivity impacts of health care investment take on increased significance. There are implications for governments in considering preventive health policy interventions as a means of increasing economic productivity, as opposed to the more traditional labour market or taxation policies.

## Conclusions

The results we present here highlight the extent of the productivity gains to society that can be achieved through investment in a junk food tax to prevent obesity. This information can be used by decision makers interested in improving both health and economic outcomes simultaneously.

## Supporting information

S1 Table Working years gained by age and sex. Dataset containing 100 bootstraped estimates of working years gained from premature deaths averted due to a junk food tax, by age and sex.
(XLSX)

S2 Table Present value of lifetime income gained by age and sex. Dataset containing 100 bootstraped estimates of present value of lifetime income gained from premature deaths averted due to a junk food tax, by age and sex.
(XLSX)

S3 Table Present value of lifetime income gained by cause of death. Dataset containing 100 bootstraped estimates of present value of lifetime income gained from premature deaths averted due to a junk food tax, by cause of death.
(XLSX)
